# Risk factors for deep surgical site infections following orthopedic trauma surgery: a meta-analysis and systematic review

**DOI:** 10.1186/s13018-024-05299-2

**Published:** 2024-11-30

**Authors:** Huan Liu, Yanan Wang, Hao Xing, Zhengqi Chang, Junlin Pan

**Affiliations:** 1 Department of Orthopedics, The 960, Hospital of PLA, 25 Shifan Road, Tiangiao District, Jinan, 250031 Shandong China; 2 Department of Reproductive Medicine, The 960, Hospital of PLA, 25 Shifan Road, Tiangiao District, Jinan, 250031 Shandong China; 3 School of Clinical Medicine, Shandong Second Medical University, Weifang, 261053 Shandong China

**Keywords:** Orthopaedic trauma, Surgical site infection, Risk factors, Meta-analysis, Fracture

## Abstract

**Objective:**

We conducted this meta-analysis to explore the risk factors and incidence of deep surgical site infections (DSSIs) after orthopaedic trauma surgery.

**Methods:**

A systematic search was conducted across various databases, including MEDLINE, Embase, Cochrane Library, and Web of Science, for studies examining risk factors associated with DSSIs after bone trauma procedures. The search concluded on September 1, 2024. Data analysis was performed using Stata 15.0.

**Results:**

Among 2,722 publications screened, 16 studies that met the eligibility criteria were included in the analysis, involving a total of 22,318 patients, of whom 894 experienced DSSIs. The meta-analysis revealed a combined incidence of deep infections after orthopedic trauma surgery at [ES = 6.7%, 95% CI (5.1%–8.2%)]. Risk factors identified included male gender [OR = 1.99, 95% CI (1.39, 2.86), p < 0.0001], current smoking status [OR = 2.60, 95% CI (1.85, 3.65), p < 0.0001], open injuries [OR = 3.17, 95% CI (1.72, 5.85), p < 0.0001], a BMI greater than 26.0 kg/m^2^ [OR = 1.95, 95% CI (1.24, 3.07), p = 0.004], wound class ≥ 2 [OR = 2.40, 95% CI (1.56, 3.70), p < 0.0001], and a surgery duration of 60 min or more [OR = 2.41, 95% CI (1.63, 3.55), p < 0.0001]. These factors significantly contribute to the risk of developing DSSIs post-surgery. However, age did not exhibit a significant difference.

**Conclusion:**

This study identifies key risk factors for DSSI following orthopedic trauma surgery, addressing a gap in the existing literature and offering some insights for clinical decision-making. To mitigate the risk of DSSI, clinical practice should encourage patients to lose weight and quit smoking prior to surgery, optimize surgical procedures, and improve wound management strategies. Future research should aim to standardize follow-up durations and further refine the classification of risk factors, in order to validate and expand on the conclusions of this study.

**Supplementary Information:**

The online version contains supplementary material available at 10.1186/s13018-024-05299-2.

## Introduction

Bone trauma surgery is a critical intervention for addressing severe fractures and other bone injuries. However, one of the significant complications associated with these procedures is Surgical Site Infections (SSIs), particularly DSSIs [1]. In the field of orthopaedic surgery, SSIs may occur within 30 days of surgery in the absence of fixation devices, or within one year in cases where a metal implant remains in place [2]. DSSIs are refer to describe infections that have penetrated the fascia or muscle layers [3]. DSSIs not only leads to considerable physical discomfort but also increases healthcare costs and negatively impacts functional recovery [4, 5]. For instance, research by Gitajn et al. [6], indicated that the Veterans RAND 12-Item Health Survey's Physical Health Score (VR-12 PCS) at six months after surgery was 3.3 points lower for patients who experienced DSSIs compared to those who did not. Furthermore, an economic analysis revealed that the medical expenses for patients with DSSIs were approximately £1,577.11 higher than for those without such infections six months following surgery [7].

Proactively identifying risk factors and implementing timely interventions are essential strategies for preventing DSSIs effectively. This method not only alleviates patient distress but also lessens the burden on healthcare resources [8]. Nonetheless, systematic reviews and meta-analyses focusing specifically on risk factors for DSSIs following orthopaedic trauma surgery are lacking, which prompted us to conduct this study to address this gap.

Additionally, we aim for this research to establish a strong evidence-based rationale for the preventive use of NPWT (Negative Pressure Wound Therapy). NPWT has been extensively employed in the management of wounds resulting from extremity trauma. A substantial body of evidence from numerous studies has demonstrated the efficacy of NPWT in preventing surgical site infections, particularly in reducing the incidence of DSSIs following orthopaedic trauma surgery [9, 10]. However, due to the high cost of NPWT, clinicians cannot apply it to all surgical patients. Therefore, it is essential for clinicians to identify risk factors for DSSI and target prophylactic measures toward high-risk groups to reduce the risk of infection and improve patient outcomes. Previous studies have identified a variety of risk factors associated with DSSIs following orthopedic trauma surgery, including male gender, older age, diabetes, smoking, alcohol abuse, open injuries, and complex fracture types [5, 11, 12]. A thorough evaluation and meta-analysis can further clarify how these factors influence DSSI incidence, providing more precise guidance for the prophylactic use of NPWT.

This study aims to explore the incidence of DSSIs and their associated risk factors in depth through systematic evaluation and meta-analysis. We hope our findings will offer valuable insights into the preventive application of NPWT, thereby lowering healthcare costs and enhancing patient prognosis.

## Information and methods

This program was developed following the guidelines outlined in the Preferred Reporting Items for Systematic Reviews and Meta-Analyses Protocols (PRISMA [13]). The reviews will adhere to these PRISMA guidelines. In carrying out this study, we adhered completely to the PRISMA guidelines without any deviations.

### Literature search

A comprehensive search strategy is detailed in Supplementary Material 1. We conducted a literature search using the MEDLINE, Embase, Cochrane Library, and Web of Science databases to identify studies focused on surgical site infections and their associated risk factors in the context of orthopedic trauma, with a search cutoff date of September 1, 2024. The literature review included both free-text terms and subject terms, such as surgical site infections, orthopedic trauma, fracture, and risk factors.

### Inclusion and exclusion criteria

Inclusion criteria: 1. The study population comprised patients who experienced DSSIs after orthopedic trauma surgeries. 2. The diagnosis of DSSIs were established [14]. The primary outcome measure was multifactorial analysis, while the secondary outcome measure was the occurrence of DSSIs post bone trauma surgery.

Exclusion criteria included: 1. Studies not meeting the deep surgical site infection diagnostic criteria. 2. Analyses that did not focus on DSSIs. 3. Studies where the study population consisted of patients undergoing total joint replacements (Total joint replacement is often considered to be the field of joint surgery and therefore performed to exclude). 4. Conference protocols, correspondence, duplicate publications, inaccessible research for original articles, etc.

**Fig. 1:**
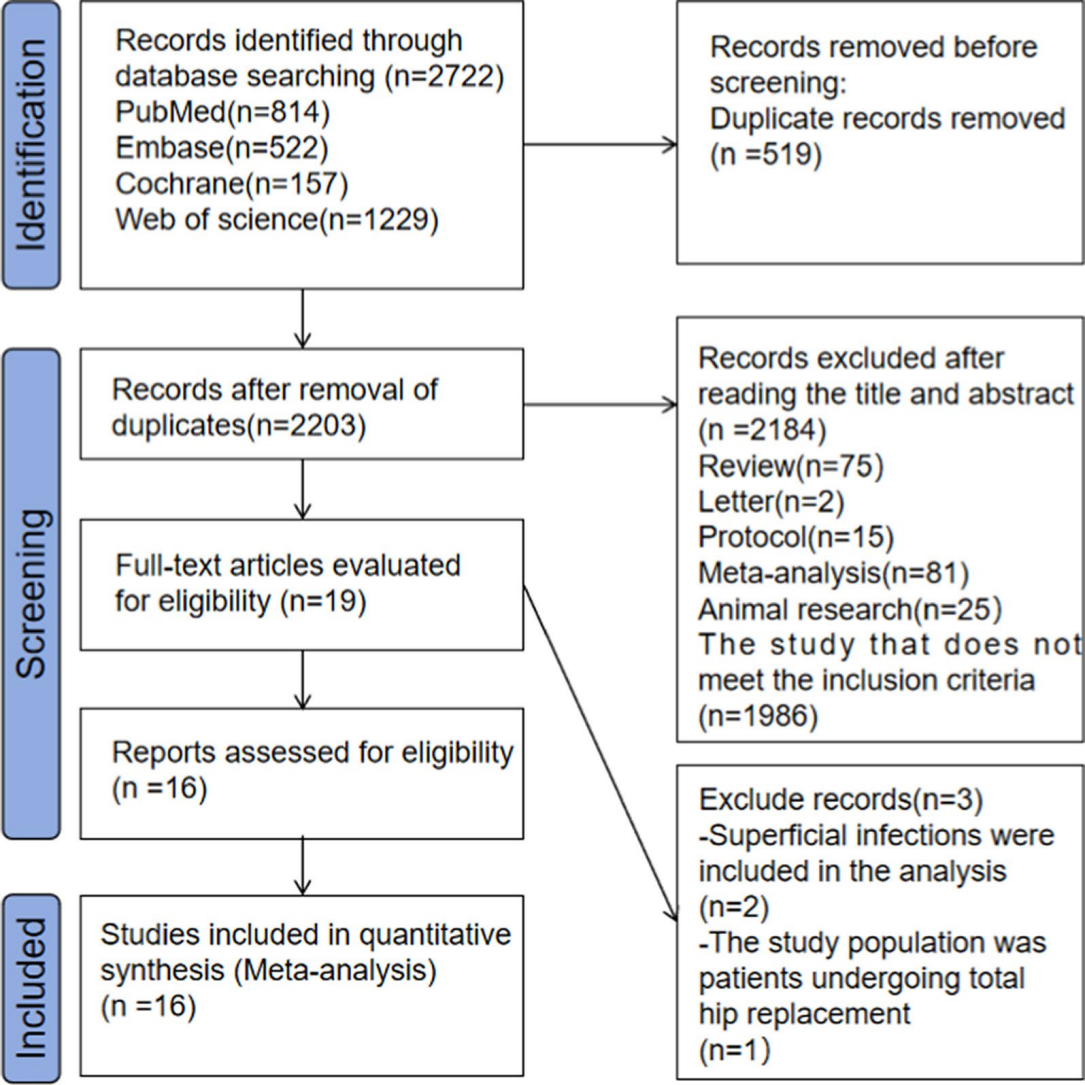
Details of the literature search and screening process

The selection of studies was not limited by size or type, and there were no restrictions on language. Figure 1 presents the details of the literature search and screening process.

### Data extraction

The first and second authors independently reviewed the literature for data extraction by examining titles, abstracts, and full texts of articles, and by consulting relevant experts regarding potentially includable literature. During screening, we adhered strictly to the established inclusion and exclusion criteria, extracting the relevant indicators from the studies and cross-referencing the data to confirm consistency. Key data extracted included: first author's name, publication year, country, study design, gender, age, sample size, and follow-up duration. The data were thoroughly extracted and cross-checked to ensure consistency.

### Quality assessment of included studies

The quality of the included studies was assessed using the Newcastle–Ottawa Scale (NOS) [15]. Each study was scored according to specific criteria: STUDY POPULATION SELECTION contributed 4 points, GROUP COMPARABILITY contributed 2 points, and EXPOSURE FACTORS OR OUTCOME MEASURES contributed 3 points. Scores ranged from 0 to 9, with studies scoring ≥ 7 classified as high quality, scores ≤ 4 as low quality (indicating exclusion), and scores between 5 and 6 as medium quality.

### Statistical analyses

If three or more studies performed multifactorial analyses on a risk factor, it was included in the meta-analysis. The odds ratios (ORs) and 95% confidence intervals for pooled risk factors were calculated with Stata 15.0. The choice of model for pooled ORs was determined by the heterogeneity test results (Q-test method) and the I^2^ statistic. A fixed-effects model was applied when I^2^ ≤ 50%. Conversely, a random effects model was used when I^2^ > 50%, with sensitivity assessed through a case-by-case exclusion test. Incidence of DSSIs was combined using the metan command in stata 15.0. Publication bias in the meta-analysis was evaluated using the Egger test and Begg test, indicating a low likelihood of bias if P > 0.05.

## Results

### Literature search and process

A search of databases including MEDLINE, Embase, the Cochrane Library and Web of Science was conducted to identify studies investigating risk factors for surgical site infections following orthopaedic trauma surgery. The management of the literature was facilitated by EndNote 21. A total of 2,722 documents were retrieved, and 2,203 were obtained after the removal of 519 duplicates. Based on the established inclusion and exclusion criteria, 19 pertinent studies were initially identified after a review of the titles and abstracts. The full texts of these studies were then evaluated to exclude two studies [15, 16] that analyzed superficial SSIs and one study [17] that included patients undergoing total joint replacement. This process resulted in the inclusion of 16 eligible papers.

### Basic characteristics of the included literature

A total of 16 [1, 4, 5, 11, 12, 18–28]studies were included, comprising 4 case–control studies and 12 cohort studies. In total, 22,318 patients were included, of whom 894 developed DSSIs. Basic information regarding the literature is summarized in Table 1. The 16 studies included in this review were evaluated using the Newcastle–Ottawa Scale (NOS), revealing that all studies scored between 7 and 9, indicating a high overall quality of inclusion. The evaluation results for each study can be found in Table 2.

**Table 1:** Characteristics of literature table

Study	Country	Design	Sample size		Gender (M/F)		Age (years)		Follow-up time	Type of surgery
			S	Total	S	NS	S	NS		
Ovaska 2013	Finland	Case control	131	1923	846/1077		56		NA	Ankle Fractures
Ma 2018	China	Case control	17	676	465/211		46.9	44.4	NA	Open reduction and internal fixation of closed tibial plateau fractures
Meng 2018	China	Case control	74	2617	39/35	2543/1482	50.6	44.2	NA	Ankle fractures treated by open reduction and internal fixation
Zhao 2022	China	Case control	74	2147	1575/572		39	41	NA	Peri-ankle fractures
Yokoyama2006	Japanese	Cohort study	7	42	35/7		25.7	33.1	from 2 to 12 years	Secondary intramedullary nailing after external fixation foropen tibial fractures
Harrison2012	UK	Cohort study	50	6905	11/39	1499/5356	79.9	81.3	1 years	Hip fracture surgery
Metsemakers 2015	Belgium	Cohort study	7	480	338/142		39.2		Minimum follow-up was 18 months	Intramedullary nailing of tibial shaft fractures
Spitler 2020	USA	Cohort study	25	150	68/57	16/9	44.2	44	fracture union or deep infection	AO/OTA 43C Pilon Fractures
Ukai 2020	Japanese	Cohort study	18	114	NA		44.5		≥ 2 years	Lower limb Gustilo–Anderson type III fractures
Anderson2021	USA	Cohort study	128	465	329/136		41.7		90 days	Pelvic, or acetabular fracture and requiring open reduction and internal fixation or intramedullary nailing
Henkelmann 2021	Germany	Cohort study	94	2106	62/32	1082/929	55	50	NA	Tibial plateau fractures
Lu 2022	China	Cohort study	24	900	23/1	812/64	41.5	41.9	≥ 1 year	Open reduction and internal fixation of displaced intra-articular calcaneal fracture
Zhu 2022	China	Cohort study	33	1562	906/656		50.3		1 year	Open reduction and internal fixation of closed ankle fracture
Brodke et al. 2023	USA	Cohort study	79	1107	450/657		57		Median follow-up was 47 weeks	OTA/AO 33A or C distal femur fractures
Wilkinson2023	USA	Cohort study	97	769	76/21	523/149	45	35	NA	Open tibial shaft fractures treated with intramedullary nail

*S* People with deep SSI; *NA* Not available

**Table 2:** Newcastle–Ottawa Scale (NOS) for studies

Study	Selection	Comparability	Outcome	Total score
Cohort study				
Yokoyama 2006	****	**	***	9
Harrison 2012	****	**	***	9
Metsemakers 2015	****	**	***	9
Spitler 2020	****	**	***	9
Ukai 2020	****	**	***	9
Anderson 2021	****	**	**	8
Henkelmann 2021	****	**	*	7
Lu 2022	****	**	***	9
Zhu 2022	****	**	***	9
Brodke 2023	****	**	***	9
Wilkinson 2023	****	**	*	7

Study	Selection	Comparability	Exposure	Total score
Case–control study				
Vaska 2013	****	**	***	9
Ma 2018	****	**	***	9
Meng 2018	****	**	***	9
Zhao 2022	****	**	***	9

## *Meta*-analysis of risk factors

### Males

**Fig. 2:**
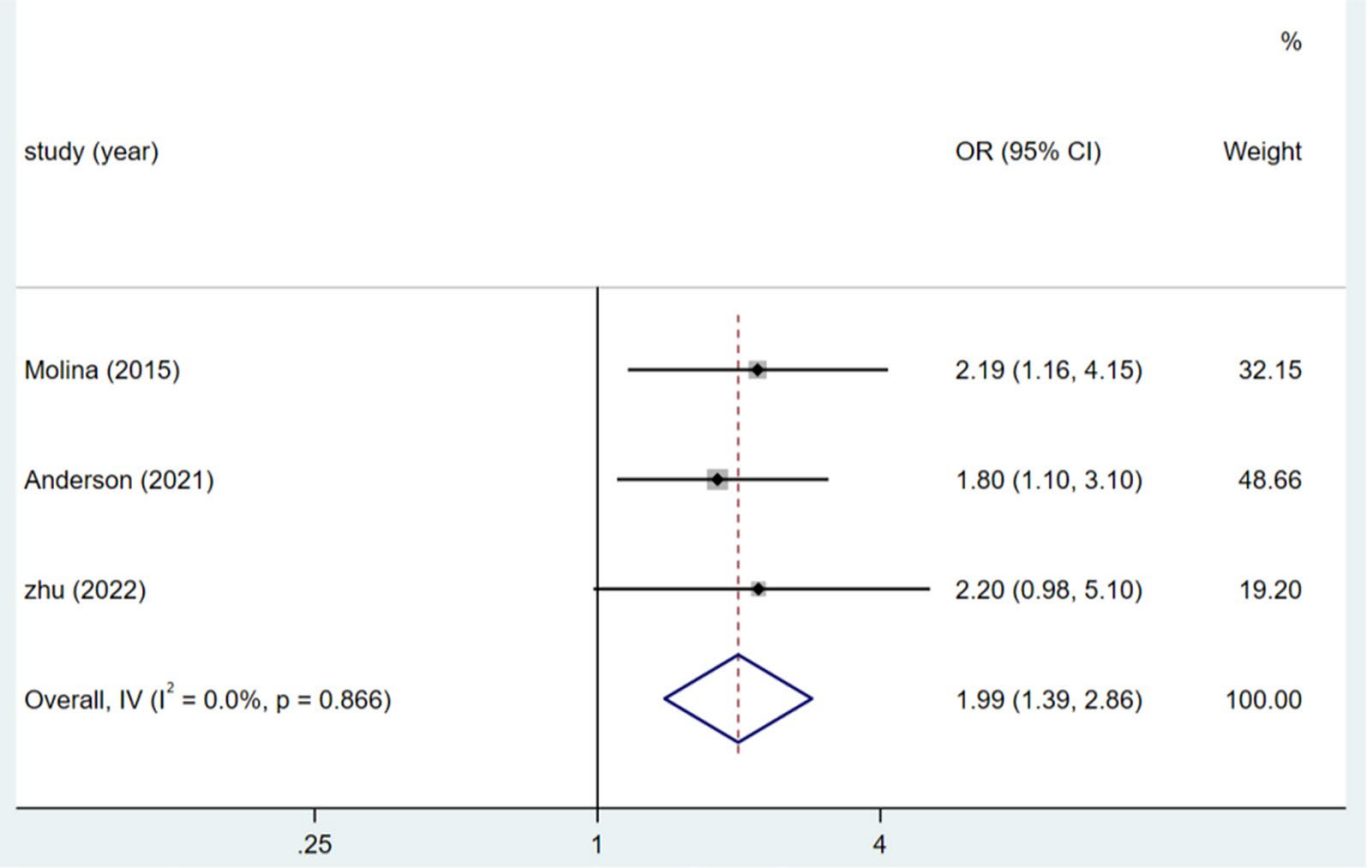
Forest plot of males as risk factors

Three studies focused on males, and the heterogeneity test (I^2^ = 0.0%, P = 0.866) was analysed using a fixed-effects model. Male gender was a risk factor for DSSIs following orthopaedic trauma surgery (OR = 1.99, 95% CI (1.39, 2.86), P < 0.0001) (see Fig. 2, Table 3).

**Table 3:** Meta-analysis

Risk factors	No. of study	heterogeneity		ES (95%CI)	P	Egger	Begg
		I^2^(%)	P				
Males	3	0.0	0.866	1.99,(1.39,2.86)	< 0.0001	0.373	1.000
BMI > 26.0 kg/m^2^	3	0.0	0.412	1.95(1.24,3.07)	0.004	0.741	1.000
Current somking	6	0.0	0.753	2.60(1.85,3.65)	< 0.0001	0.409	0.230
Wound class ≥ 2	3	38.7	0.196	2.40(1.56,3.70)	< 0.0001	0.199	1.000
Duration of surgery ≥ 60 min	5	0.0	0.662	2.41(1.63, 3.55)	< 0.0001	0.622	0.806
Open injury	5	81.1	< 0.001	3.17(1.72, 5.85)	< 0.0001	0.980	0.902

### BMI

**Fig. 3:**
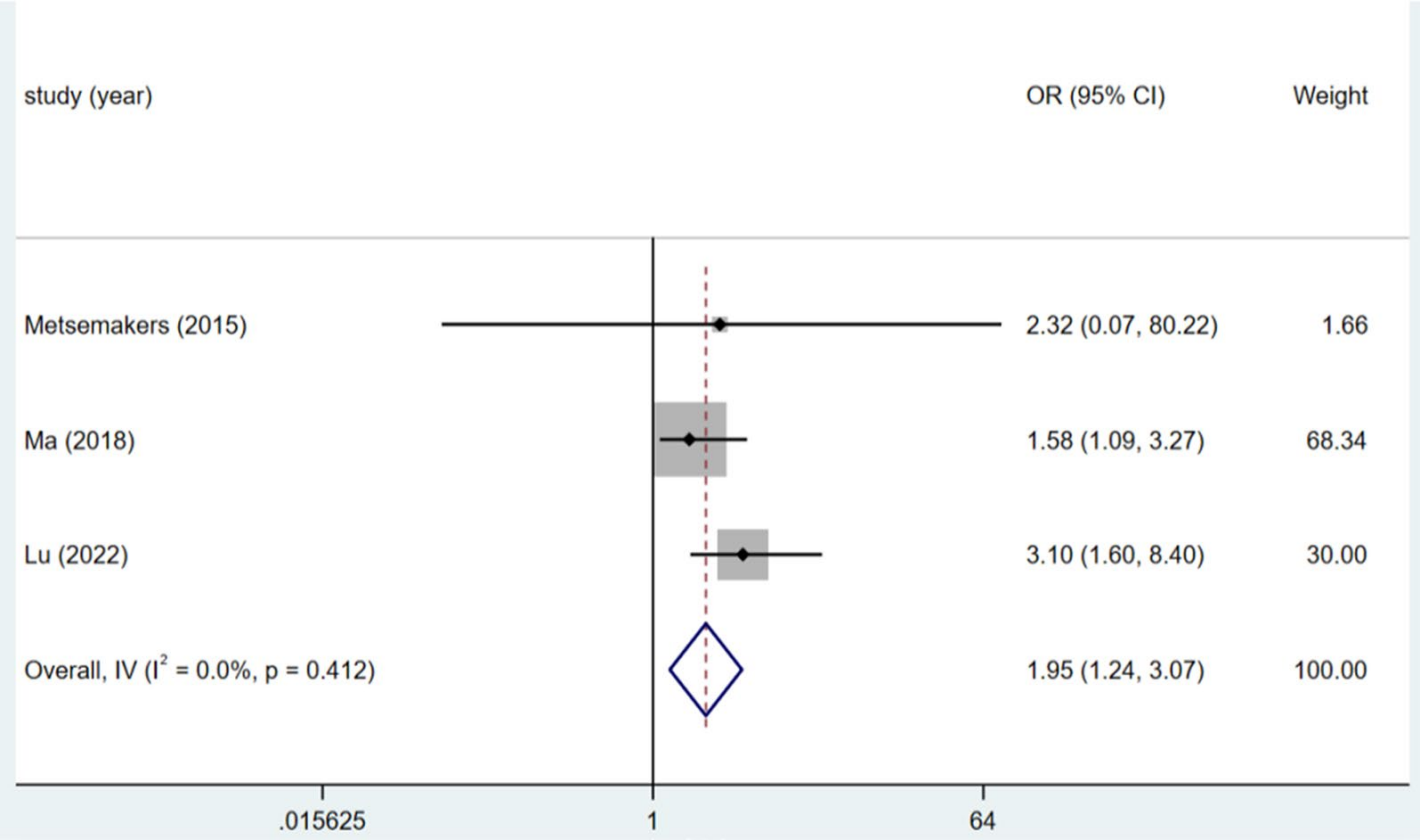
Forest plot of BMI > 26.0 kg/m^2^ as risk factors

The results of three studies on BMI were analysed using a fixed-effects model, as the heterogeneity test (I^2^ = 0.0%, P = 0.412). A BMI greater than 26.0 kg/m^2^ was a risk factor for DSSIs following bone trauma surgery (OR = 1.95, 95% CI (1.24, 3.07), P = 0.004).(see Fig. 3, Table 3).

### Current somking

**Fig. 4:**
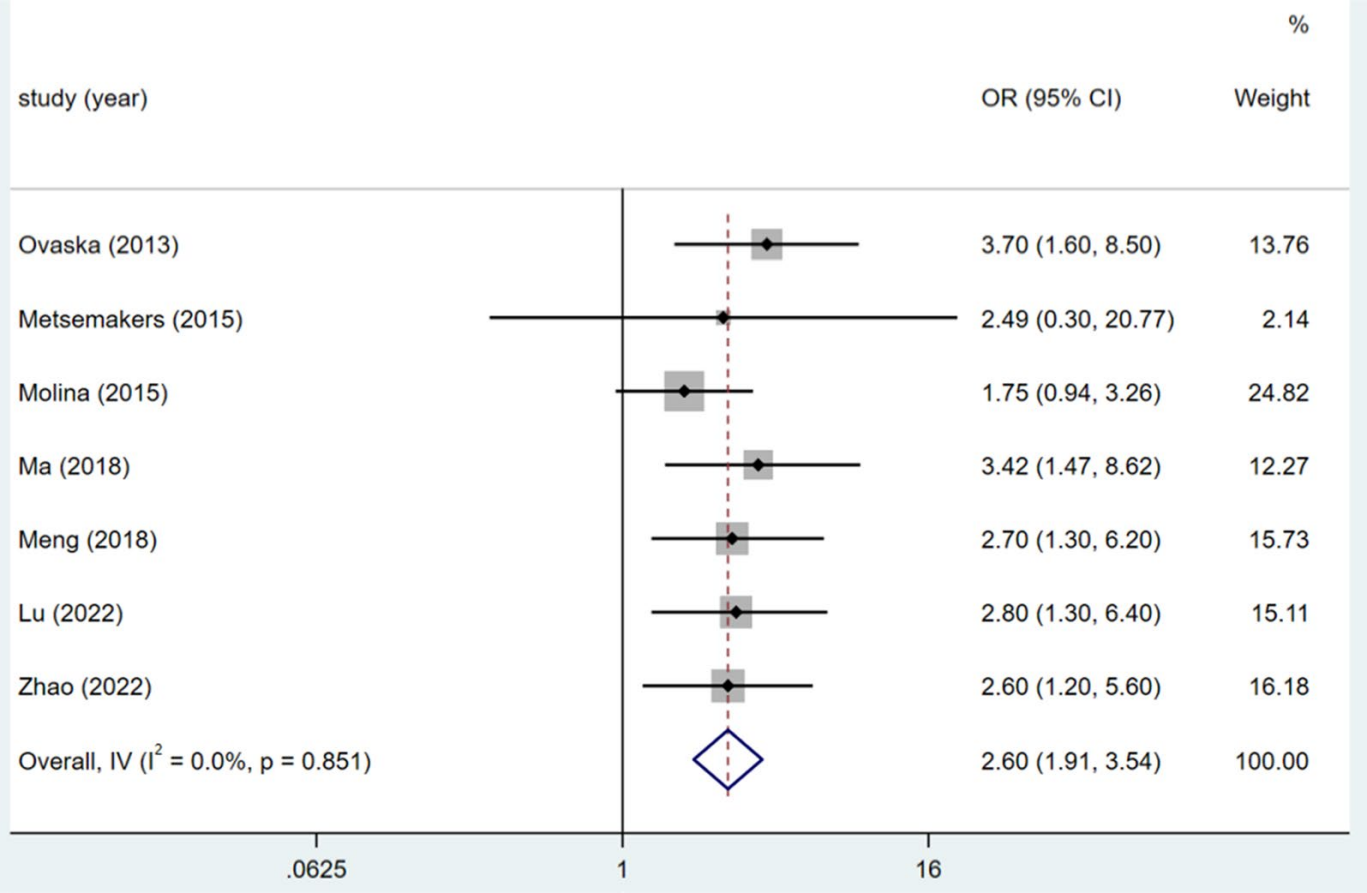
Forest plot of current somking as risk factors

Six studies addressed the topic of current smoking, and the heterogeneity test (I^2^ = 0.0%, P = 0.753) was analysed using a fixed-effects model. Current smoking increases the risk of DSSIs following orthopaedic trauma surgery (OR = 2.60, 95% CI (1.85, 3.65), P < 0.0001).(see Fig. 4, Table 3).

### Wound class

**Fig. 5:**
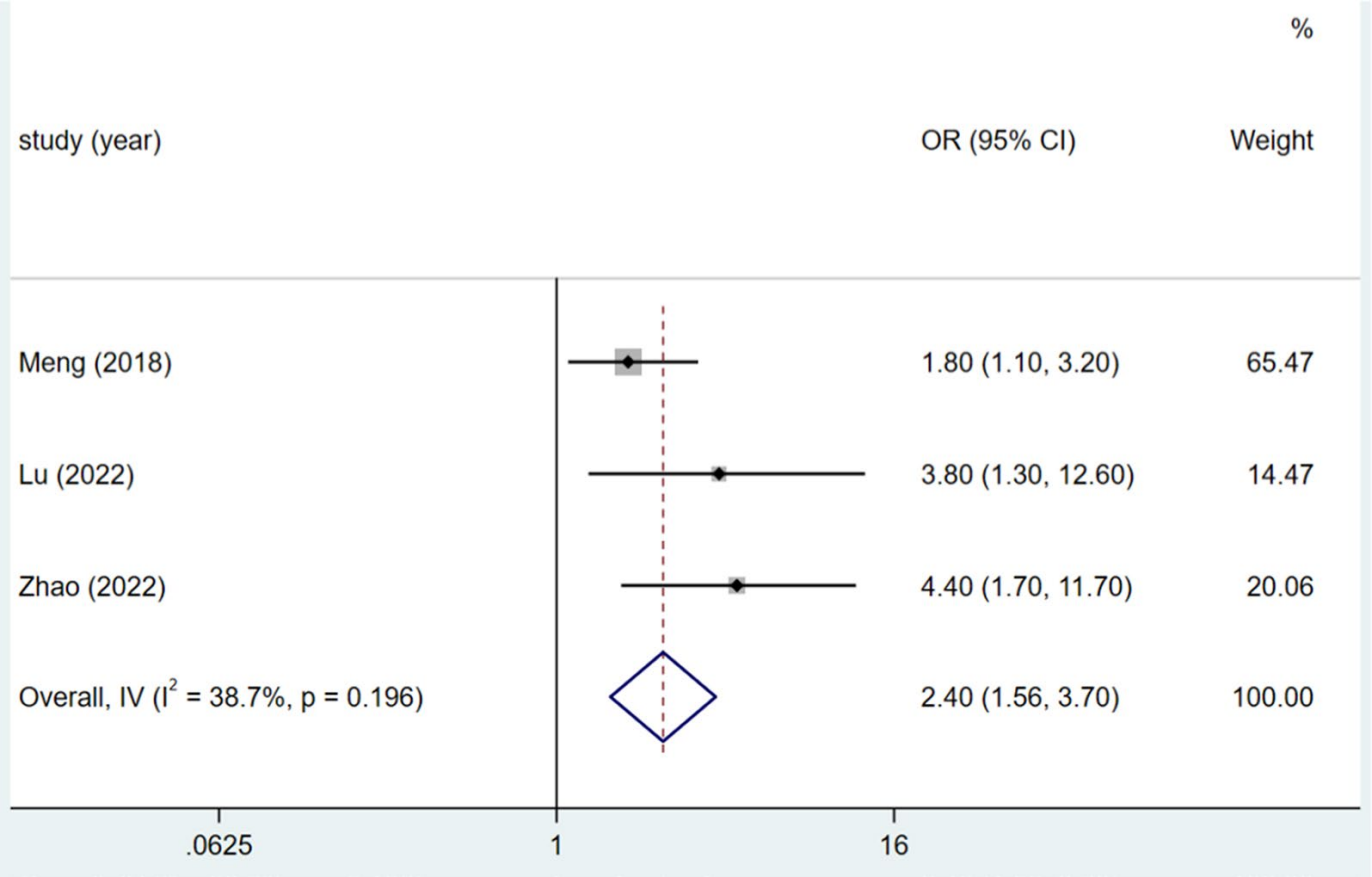
Forest plot of wound class ≥ 2 as risk factors

Three studies addressed the classification of wounds, and the heterogeneity test (I^2^ = 38.7%, P = 0.196) was analysed using a fixed-effects model. A wound class of 2 or above was a significant risk factor for DSSIs following orthopaedic trauma surgery (OR = 2.40, 95% CI (1.56, 3.70), P < 0.0001).(see Fig. 5, Table 3).

### Duration of surgery

**Fig. 6:**
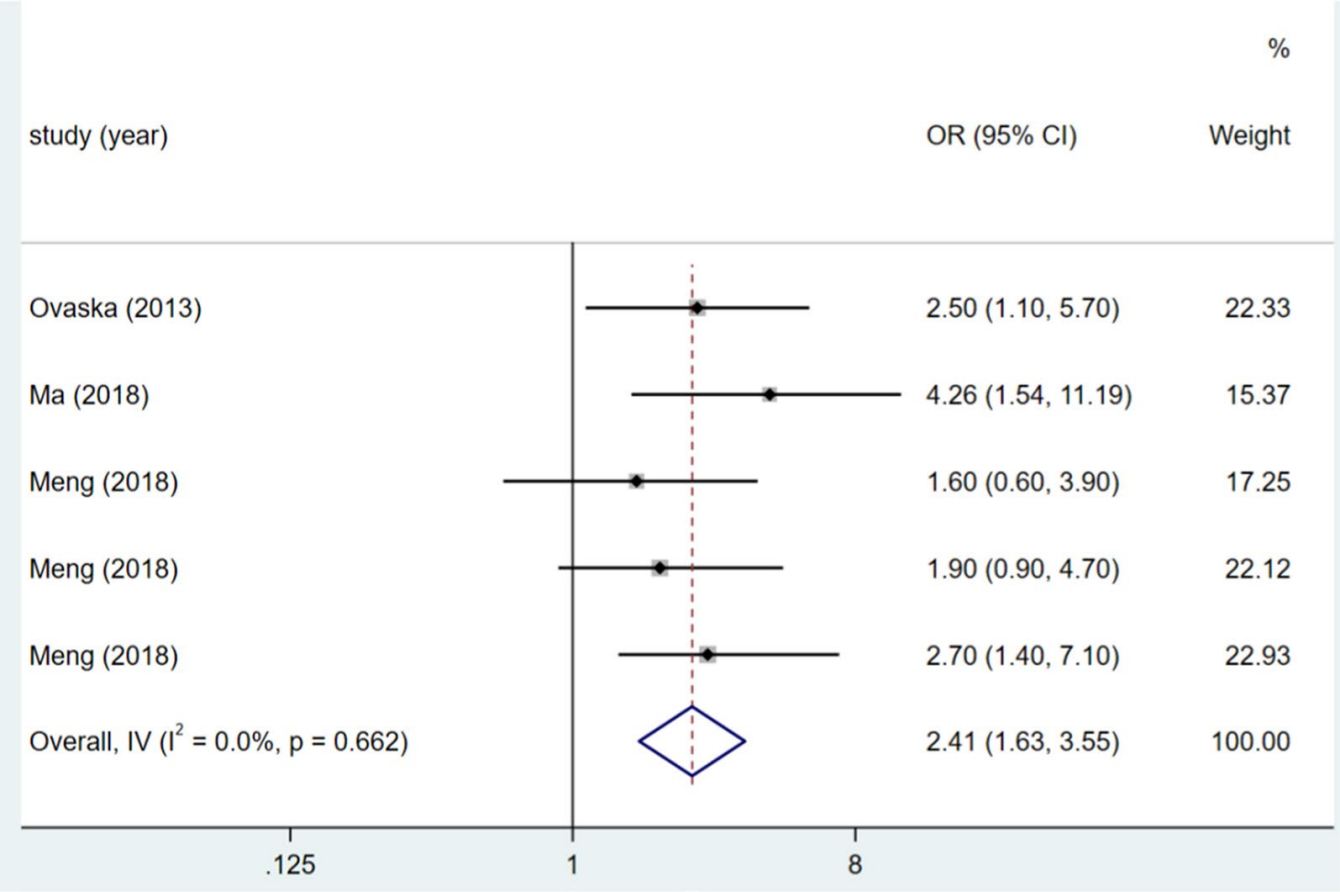
Forest plot of duration of surgery ≥ 60 min as risk factors

The duration of surgery was examined in five studies, and the heterogeneity test (I^2^ = 0.0%, P = 0.662) was analysed using a fixed-effects model. A duration of surgery exceeding 60 min was a risk factor for DSSIs following bone trauma surgery (OR = 2.41, 95% CI (1.63, 3.55), P < 0.0001).(see Fig. 6, Table 3).

### Open injury

**Fig. 7:**
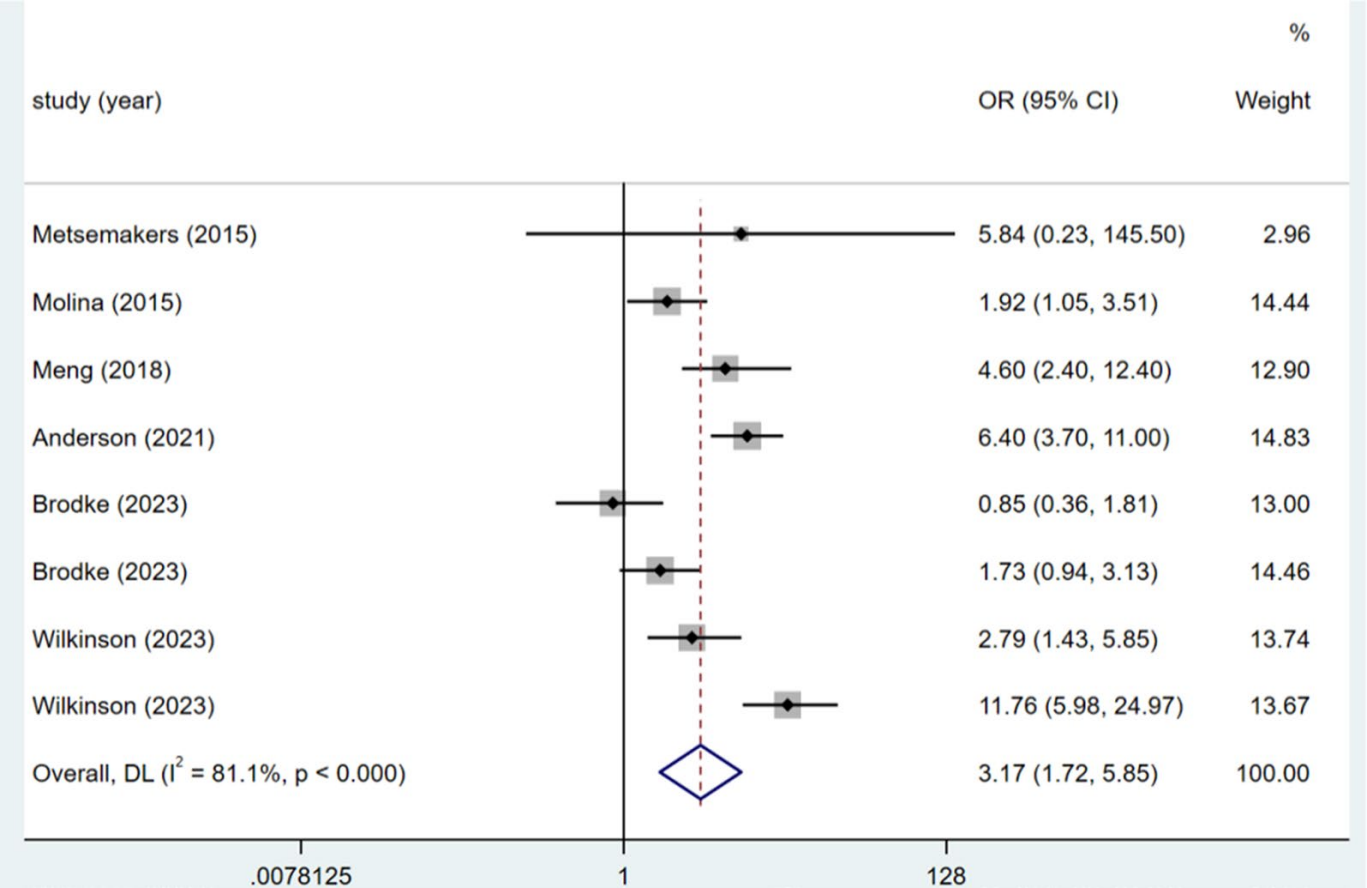
Forest plot of open injury as risk factors

Five studies reported on open injuries, and the heterogeneity test (I^2^ = 81.1%, P < 0.001) was analysed using a random-effects model. Open injuries raise the risk of DSSIs following orthopaedic trauma surgery (OR = 3.17, 95% CI (1.72, 5.85), P < 0.0001) (see Fig. 7, Table 3).

## Combined incidence of DSSIs after bone trauma surgery

A total of 16 studies were identified that reported on the incidence of DSSIs following orthopaedic trauma surgery. The heterogeneity test (I^2^ = 97.5%, P < 0.001) was analysed using a random-effects model. The meta-analysis indicated an incidence of DSSIs after orthopaedic trauma surgery of [ES = 6.7%, 95% CI (5.1%–8.2%)]. Due to the significant heterogeneity caused by comorbidities, each study was excluded one by one for sensitivity analysis. The results suggested that the sensitivity was low and the findings were relatively stable. The Egger test was performed to evaluate publication bias for this indicator, yielding P < 0.001, indicating a higher likelihood of publication bias (see Supplementary Material 2).

## Sensitivity analyses

Sensitivity analyses were conducted by omitting one study at a time to assess its impact on the pooled results. The results of the analyses showed that the pooled results for the risk factors and their 95% confidence intervals were not significantly affected by any of the individual studies, with the exception of BMI and wound grade.(For detailed information, please refer to Supplementary Material 3).

## Publication *bias*

Egger test and Begg test results indicated the following: male (Egger: P = 0.373,Begg: P = 1.000), BMI > 26.0 kg/m^2^ (Egger: P = 0.741,Begg: P = 1.000), current smoking (Egger: P = 0.409,Begg: P = 0.230), wound class ≥ 2 (Egger: P = 0.199,Begg: P = 1.000), duration of surgery ≥ 60 min (Egger: P = 0.622,Begg: P = 0.806), and open injury (Egger: P = 0.980,Begg: P = 0.902). All P-values were > 0.05, suggesting a low possibility of publication bias.

## Discussion

This meta-analysis examines the incidence and risk factors associated with DSSIs after bone trauma surgery. The overall incidence of DSSIs identified in the study was 6.7%. Factors such as male gender, BMI > 26.0 kg/m^2^, current smoking, wound class ≥ 2, duration of surgery ≥ 60 min, and open injury were found to increase the risk of DSSIs post-bone trauma surgery, while age did not demonstrate a significant effect.

There are some limitations to this study that could impact the interpretation and generalization of the results. Firstly, a majority of the studies included were of a retrospective nature, which could introduce information bias. These studies often relied on pre-existing medical records and patient self-reports, which may result in incomplete or inaccurate data, potentially underestimating or overstating certain risk factors. Secondly, the total number of studies included in our analysis was relatively small, particularly when risk factors were examined individually. The insufficient sample size might reduce the statistical power and affect the reliability of the findings. Moreover, due to the absence of data regarding patients with diabetes mellitus and those with a higher ASA classification, these commonly recognized risk factors were not incorporated into our analysis, further limiting the broader applicability of the results.

Despite these limitations, we are confident in the main conclusions of our study. This confidence stems from the consistency and overall trends observed across the included studies, which highlighted that factors such as male gender, elevated BMI, smoking, and open wounds were strongly associated with the occurrence of postoperative DSSIs. Additionally, we employed a rigorous methodology for data collection and analysis to minimize bias, specifically by including only the results of multifactorial analyses across studies to control for confounding variables. While the retrospective nature of the design and missing data may have impacted some aspects, we believe our research maintains a high level of credibility and provides valuable guidance for clinical practice. Future studies should address these challenges to further corroborate and expand on our findings.

Concerning the combined morbidity, this meta-analysis revealed significant heterogeneity, primarily linked to the variety of orthopedic surgical types and differences in follow-up duration. Although the included studies focused on orthopedic trauma, there were notable variations in surgical complexity, the degree of trauma, and the surgical approach across different types of trauma surgeries, all of which impacted the incidence of postoperative DSSIs. Additionally, the follow-up duration significantly contributed to this heterogeneity. Theoretically, longer follow-up times are associated with a higher likelihood of detecting postoperative deep SSIs, as infections may emerge later. However, the studies varied widely in their follow-up periods, with some extending only a few months while others lasted several years, further exacerbating heterogeneity. To mitigate this in future studies, it is recommended to standardize follow-up durations. For instance, uniformly tracking patients for at least one year would enhance the detection of postoperative infections and yield more comparable data. Establishing such a follow-up standard not only improves data comparability but also provides a reliable foundation for future meta-analyses, allowing for subgroup analyses based on follow-up time, thus reducing heterogeneity across studies.

Regarding the demographic characteristics of the patients, the results of the multifactorial analysis of 3 studies [12, 20, 24] involved males, and the results of the studies showed that males were a risk factor for DSSIs after bone trauma surgery. Regarding age, the multifactorial analysis of 5 studies [11, 12, 23–25]reported OR values and 95% confidence intervals, and we analysed the relevant data, and the findings showed no significant difference [OR = 1.077,95% CI (0.998, 1.162), P = 0.056]. However, this may require further explanation as only two [11, 23] of the five studies included in the analysis were stratified for age. When 40 years of age was selected as the threshold to include the results of two studies in the analysis, the findings showed a significant difference, with age > 40 years increasing the incidence of DSSIs after bone trauma surgery[OR = 2.44,95% CI (1.79, 3.34), P < 0.0001]. As there were only two studies, we did not describe the results in the meta-analysis section. Future studies may consider stratifying the age analysis or using ROC curves to obtain an appropriate threshold when performing similar analyses, which would provide more data for similar meta-analyses and further clarify the effect of age on DSSIs.

Higher BMI and smoking have been identified as independent factors contributing to surgical site infections in numerous studies, strongly linked to a poor wound environment resulting from both [19, 29, 30]. As BMI increases, the thickness of subcutaneous fat also increases, potentially leading to local hypoxia at the incision due to poor fat vascularization and decreased oxygen tension [31]. This lack of oxygen impedes neutrophils from effectively phagocytosing bacteria, subsequently raising the risk of postoperative infections [32]. Furthermore, thicker subcutaneous fat prolongs exposure and retraction of the surgical area, resulting in greater soft tissue damage [33]. It also contributes to localized dead space formation and reduces tissue penetration of antibiotics [31, 34]. These factors negatively impact wound healing, consequently increasing the risk of postoperative infection. Smoking significantly diminishes tissue blood flow, oxygen tension, and aerobic metabolism [35]. The hypoxic environment weakens the immune response within the wound and slows healing, further heightening the risk of infection [29, 36]. Studies have indicated that smoking reduces inflammatory cell infiltration and macrophage counts in wounds while diminishing neutrophil defense against pathogens [37, 38]. Additionally, smoking impairs fibroblast function and inhibits collagen synthesis, adversely affecting the proliferative phase of wound healing and the tissue remodeling process [37]. Although the sensitivity analyses of BMI are not robust, we remain confident in our main conclusions, as numerous previous studies have demonstrated that higher BMI increases the risk of surgical site infections following orthopaedic trauma surgery [39–43]. The small number of studies included in the analysis means that a single study can significantly impact the sensitivity analysis results. However, we believe that as more data are incorporated into future analyses, the sensitivity analysis results will stabilize, and the combined findings will continue to support the conclusion that higher BMI is associated with an increased risk of DSSI.

Longer surgical times and open injuries indicate more complex surgical operations, resulting in prolonged wound exposure and greater trauma to the patient, which fosters conditions conducive to bacterial invasion [44, 45]. Open injuries further elevate infection risk due to direct exposure of the wound to the external environment. Two included studies [5, 11] utilized Gustilo-Anderson typing for open injuries, and our analysis indicated that Gustilo-Anderson type III was a significant risk factor for DSSIs [OR = 3.80, 95% CI (1.24, 11.67), P < 0.0001]. There was significant heterogeneity in the results related to open injuries. This may stem from the inclusion of various types of open injuries without adequate differentiation and stratification in the studies, contributing to the significant heterogeneity of results. Previous studies have shown that Gustilo-Anderson type III fractures have a higher infection rate compared to type I and II fractures [46]. Consequently, when one study predominantly included type I and type II fractures with low infection rates while another included a substantial number of type III fractures with high infection rates, the difference in patient groups could lead to inconsistent overall results. Future research should carefully classify open injuries according to Gustilo et al.’s criteria [46] to further clarify the impact of different open injuries on DSSIs.

The risk of surgical site infections are closely related to the grade of wound contamination, with higher levels of contamination correlating with an increased risk of infection [47]. As wound contamination rises, the likelihood of pathogen invasion into the surgical site significantly increases. In clean wounds, the infection risk remains relatively low due to strict aseptic practices that limit pathogen entry. However, in contaminated or infected wounds, pathogen load increases significantly, leading to a substantial rise in the risk of SSIs. According to Delgado-Rodríguez et al. [48]the infection rate for dirty/infected wounds was as high as 2.8%, three times higher than for clean-contaminated wounds (1%) and clean wounds (0.9%), and also higher than for contaminated wounds (1.8%). Therefore, it is critical to implement targeted infection prevention and control strategies for various wound types based on the CDC wound classification [49]. For instance, aseptic practices should be intensified for clean wounds; prophylactic antibiotics should be administered for clean-contaminated wounds; and more aggressive anti-infective treatments should be considered for contaminated and dirty/infected wounds, with extended monitoring of patients. Although the results of the sensitivity analyses for wound class were not stable, the lack of significant changes in the findings for higher wound class as a risk factor after omitting each study individually did not undermine our main conclusions. We believe that the sensitivity analyses for wound class will stabilize with the inclusion of more studies in the future.

The risk factors identified in this study are already known to the orthopaedic community. Numerous studies have demonstrated a correlation between these factors and the incidence of postoperative infections, aligning our research with evidence-based practice. Our findings offer more specific quantitative data regarding these established factors, which will assist clinicians in evaluating the risk of deep-seated infections for individual patients and implementing effective preventive strategies. In particular, since patients with elevated BMI and those who smoke are at an increased risk of infection, it is crucial to encourage weight loss and smoking cessation prior to surgery, along with providing appropriate support. Additionally, the negative impact of prolonged operative times highlights the need to optimize surgical protocols. The challenges related to infection risk and wound management in higher wound grades and open injuries make the use of prophylactic NPWT a worthwhile consideration.

## Conclusion

Our analysis indicates that factors such as male gender, elevated BMI, higher wound grade, extended operative time, current smoking status, and open wounds significantly contribute to the risk of DSSIs following orthopaedic trauma surgery. It is essential for clinicians to closely monitor these indicators and consider implementing preventive strategies. In clinical practice, patients should be encouraged to lose weight and quit smoking prior to surgery, optimize surgical protocols, and improve wound management. Prospective studies with large sample sizes are still needed in the future, emphasizing standardized follow-up durations and refined risk factor classifications to further validate and expand upon our findings. Additionally, further investigation is required to assess the impact of open wounds and age on the occurrence of DSSIs.

## Supplementary Information

Supplementary file 1.

## Data Availability

No datasets were generated or analysed during the current study.
