# Supplementary material for: Risk factors for deep surgical site infections following orthopedic trauma surgery: a meta-analysis and systematic review
Source: J Orthop Surg Res. 2024 Nov 30;19:811. doi: 10.1186/s13018-024-05299-2 (PMC11607913; doi:10.1186/s13018-024-05299-2)

**Supplementary Material 1: Search Record**

**Supplementary Material 2: Supplementary Material 2: Forest plot of the incidence of deep surgical site infections, sensitivity analysis and egger's test**

**Supplementary Material 3:Sensitivity analyses of risk factors**

**Supplementary Material 1: Search History**

**1.PubMed**

| **Search number** | **Query** | **Results** |
| --- | --- | --- |
| **1** | **Orthopaedic trauma** | **193,456** |
| **2** | **Orthopaedic trauma surgery** | **64,969** |
| **3** | **(Orthopaedic trauma surgery) OR (Orthopaedic trauma)** | **195,785** |
| **4** | **"Surgical Wound Infection"[Mesh]** | **41,762** |
| **5** | **(((((((((((((((Surgical Wound Infection[Title/Abstract]) OR (Infections, Surgical Wound[Title/Abstract])) OR (Surgical Wound Infections[Title/Abstract])) OR (Wound Infections, Surgical[Title/Abstract])) OR (Infection, Surgical Wound[Title/Abstract])) OR (Surgical Site Infection[Title/Abstract])) OR (Infection, Surgical Site[Title/Abstract])) OR (Infections, Surgical Site[Title/Abstract])) OR (Surgical Site Infections[Title/Abstract])) OR (Wound Infection, Postoperative[Title/Abstract])) OR (Wound Infection, Surgical[Title/Abstract])) OR (Infection, Postoperative Wound[Title/Abstract])) OR (Infections, Postoperative Wound[Title/Abstract])) OR (Postoperative Wound Infections[Title/Abstract])) OR (Wound Infections, Postoperative[Title/Abstract])) OR (Postoperative Wound Infection[Title/Abstract])** | **21,773** |
| **6** | **("Surgical Wound Infection"[Mesh]) OR ((((((((((((((((Surgical Wound Infection[Title/Abstract]) OR (Infections, Surgical Wound[Title/Abstract])) OR (Surgical Wound Infections[Title/Abstract])) OR (Wound Infections, Surgical[Title/Abstract])) OR (Infection, Surgical Wound[Title/Abstract])) OR (Surgical Site Infection[Title/Abstract])) OR (Infection, Surgical Site[Title/Abstract])) OR (Infections, Surgical Site[Title/Abstract])) OR (Surgical Site Infections[Title/Abstract])) OR (Wound Infection, Postoperative[Title/Abstract])) OR (Wound Infection, Surgical[Title/Abstract])) OR (Infection, Postoperative Wound[Title/Abstract])) OR (Infections, Postoperative Wound[Title/Abstract])) OR (Postoperative Wound Infections[Title/Abstract])) OR (Wound Infections, Postoperative[Title/Abstract])) OR (Postoperative Wound Infection[Title/Abstract]))** | **51,670** |
| **7** | **"Risk Factors"[Mesh]** | **1,000,014** |
| **8** | **((((((((((((((((((Risk Factors[Title/Abstract]) OR (Factor, Risk[Title/Abstract])) OR (Risk Factor[Title/Abstract])) OR (Social Risk Factors[Title/Abstract])) OR (Factor, Social Risk[Title/Abstract])) OR (Factors, Social Risk[Title/Abstract])) OR (Risk Factor, Social[Title/Abstract])) OR (Risk Factors, Social[Title/Abstract])) OR (Social Risk Factor[Title/Abstract])) OR (Health Correlates[Title/Abstract])) OR (Correlates, Health[Title/Abstract])) OR (Population at Risk[Title/Abstract])) OR (Populations at Risk[Title/Abstract])) OR (Risk Scores[Title/Abstract])) OR (Risk Score[Title/Abstract])) OR (Score, Risk[Title/Abstract])) OR (Risk Factor Scores[Title/Abstract])) OR (Risk Factor Score[Title/Abstract])) OR (Score, Risk Factor[Title/Abstract])** | **876,514** |
| **9** | **(((((((((((((((((((Risk Factors[Title/Abstract]) OR (Factor, Risk[Title/Abstract])) OR (Risk Factor[Title/Abstract])) OR (Social Risk Factors[Title/Abstract])) OR (Factor, Social Risk[Title/Abstract])) OR (Factors, Social Risk[Title/Abstract])) OR (Risk Factor, Social[Title/Abstract])) OR (Risk Factors, Social[Title/Abstract])) OR (Social Risk Factor[Title/Abstract])) OR (Health Correlates[Title/Abstract])) OR (Correlates, Health[Title/Abstract])) OR (Population at Risk[Title/Abstract])) OR (Populations at Risk[Title/Abstract])) OR (Risk Scores[Title/Abstract])) OR (Risk Score[Title/Abstract])) OR (Score, Risk[Title/Abstract])) OR (Risk Factor Scores[Title/Abstract])) OR (Risk Factor Score[Title/Abstract])) OR (Score, Risk Factor[Title/Abstract])) OR ("Risk Factors"[Mesh])** | **1,489,169** |
| **10** | **"Fractures, Bone"[Mesh]** | **216,261** |
| **11** | **fracture[Title/Abstract]** | **230,663** |
| **12** | **("Fractures, Bone"[Mesh]) OR (fracture[Title/Abstract])** | **327,462** |
| **13** | **(("Fractures, Bone"[Mesh]) OR (fracture[Title/Abstract])) OR ((Orthopaedic trauma surgery) OR (Orthopaedic trauma))** | **443,020** |
| **14** | **(((("Fractures, Bone"[Mesh]) OR (fracture[Title/Abstract])) OR ((Orthopaedic trauma surgery) OR (Orthopaedic trauma))) AND ((((((((((((((((((((Risk Factors[Title/Abstract]) OR (Factor, Risk[Title/Abstract])) OR (Risk Factor[Title/Abstract])) OR (Social Risk Factors[Title/Abstract])) OR (Factor, Social Risk[Title/Abstract])) OR (Factors, Social Risk[Title/Abstract])) OR (Risk Factor, Social[Title/Abstract])) OR (Risk Factors, Social[Title/Abstract])) OR (Social Risk Factor[Title/Abstract])) OR (Health Correlates[Title/Abstract])) OR (Correlates, Health[Title/Abstract])) OR (Population at Risk[Title/Abstract])) OR (Populations at Risk[Title/Abstract])) OR (Risk Scores[Title/Abstract])) OR (Risk Score[Title/Abstract])) OR (Score, Risk[Title/Abstract])) OR (Risk Factor Scores[Title/Abstract])) OR (Risk Factor Score[Title/Abstract])) OR (Score, Risk Factor[Title/Abstract])) OR ("Risk Factors"[Mesh]))) AND (("Surgical Wound Infection"[Mesh]) OR ((((((((((((((((Surgical Wound Infection[Title/Abstract]) OR (Infections, Surgical Wound[Title/Abstract])) OR (Surgical Wound Infections[Title/Abstract])) OR (Wound Infections, Surgical[Title/Abstract])) OR (Infection, Surgical Wound[Title/Abstract])) OR (Surgical Site Infection[Title/Abstract])) OR (Infection, Surgical Site[Title/Abstract])) OR (Infections, Surgical Site[Title/Abstract])) OR (Surgical Site Infections[Title/Abstract])) OR (Wound Infection, Postoperative[Title/Abstract])) OR (Wound Infection, Surgical[Title/Abstract])) OR (Infection, Postoperative Wound[Title/Abstract])) OR (Infections, Postoperative Wound[Title/Abstract])) OR (Postoperative Wound Infections[Title/Abstract])) OR (Wound Infections, Postoperative[Title/Abstract])) OR (Postoperative Wound Infection[Title/Abstract])))** | **814** |

**2.Embase**

| **Search number** | **Query** | **Results** |
| --- | --- | --- |
| **1** | **'orthopedic trauma'/exp** | **24** |
| **2** | **'orthopedic trauma':ab,ti** | **1,420** |
| **3** | **'orthopedic trauma surgery':ab,ti** | **166** |
| **4** | **'fracture':ab,ti** | **271,680** |
| **5** | **#1 OR #2 OR #3 OR #4** | **272,534** |
| **6** | **'surgical infection'/exp** | **71,376** |
| **7** | **'surgical infection':ab,ti** | **1,498** |
| **8** | **'infections, surgical wound':ab,ti** | **20** |
| **9** | **'wound infections, surgical':ab,ti** | **14** |
| **10** | **'infection, surgical wound':ab,ti** | **18** |
| **11** | **'surgical site infection':ab,ti** | **17,245** |
| **12** | **'infection, surgical site':ab,ti** | **149** |
| **13** | **'infections, surgical site':ab,ti** | **110** |
| **14** | **'surgical site infections':ab,ti** | **11,891** |
| **15** | **'wound infection, postoperative':ab,ti** | **123** |
| **16** | **'wound infection, surgical':ab,ti** | **32** |
| **17** | **'infection, postoperative wound':ab,ti** | **7** |
| **18** | **'infections, postoperative wound':ab,ti** | **9** |
| **19** | **'postoperative wound infections':ab,ti** | **977** |
| **20** | **'wound infections, postoperative':ab,ti** | **39** |
| **21** | **'postoperative wound infection':ab,ti** | **1,679** |
| **22** | **#6 OR #7 OR #8 OR #9 OR #10 OR #11 OR #12 OR #13 OR #14 OR #15 OR #16 OR #17 OR #18 OR #19 OR #20 OR #21** | **76,903** |
| **23** | **'risk factor'/exp** | **1,458,013** |
| **24** | **'risk factor':ab,ti** | **421,033** |
| **25** | **'factor, risk':ab,ti** | **359** |
| **26** | **'risk factor':ab,ti** | **421,033** |
| **27** | **'social risk factors':ab,ti** | **1,482** |
| **28** | **'factor, social risk':ab,ti** | **3** |
| **29** | **'factors, social risk':ab,ti** | **10** |
| **30** | **'risk factor, social':ab,ti** | **8** |
| **31** | **'risk factors, social':ab,ti** | **154** |
| **32** | **'social risk factor':ab,ti** | **125** |
| **33** | **'health correlates':ab,ti** | **654** |
| **34** | **'correlates, health':ab,ti** | **12** |
| **35** | **'population at risk':ab,ti** | **6,119** |
| **36** | **'populations at risk':ab,ti** | **3,803** |
| **37** | **'risk scores':ab,ti** | **24,557** |
| **38** | **'risk score':ab,ti** | **51,885** |
| **39** | **'score, risk':ab,ti** | **1,379** |
| **40** | **'risk factor scores':ab,ti** | **152** |
| **41** | **'risk factor score':ab,ti** | **302** |
| **42** | **'score, risk factor':ab,ti** | **17** |
| **43** | **#23 OR #24 OR #25 OR #26 OR #27 OR #28 OR #29 OR #30 OR #31 OR #32 OR #33 OR #34 OR #35 OR #36 OR #37 OR #38 OR #39 OR #40 OR #41 OR #42** | **1,680,867** |
| **44** | **#5 AND #22 AND #43** | **522** |

**3. Cochrane library**

| **Search number** | **Query** | **Results** |
| --- | --- | --- |
| **1** | **MeSH descriptor: [Orthopedics] explode all trees** | **24863** |
| **2** | **MeSH descriptor: [Surgical Wound Infection] explode all trees** | **4623** |
| **3** | **(Surgical Wound Infections):ti,ab,kw OR (Surgical Wound Infection):ti,ab,kw OR (Infections, Surgical Wound):ti,ab,kw OR (Wound Infections, Surgical):ti,ab,kw OR (Infection, Surgical Wound):ti,ab,kw** | **9674** |
| **4** | **(Surgical Site Infection):ti,ab,kw OR (Infection, Surgical Site):ti,ab,kw OR (Infections, Surgical Site):ti,ab,kw OR (Surgical Site Infections):ti,ab,kw OR (Wound Infection, Postoperative):ti,ab,kw** | **10832** |
| **5** | **(Wound Infection, Surgical):ti,ab,kw OR (Infection, Postoperative Wound):ti,ab,kw OR (Infections, Postoperative Wound):ti,ab,kw OR (Postoperative Wound Infections):ti,ab,kw OR (Wound Infections, Postoperative):ti,ab,kw** | **11300** |
| **6** | **(Postoperative Wound Infection):ti,ab,kw** | **7061** |
| **7** | **#3 OR #4 OR #5 OR #6** | **13658** |
| **8** | **MeSH descriptor: [Risk Factors] explode all trees** | **38391** |
| **9** | **(Risk Factors):ti,ab,kw OR (Factor, Risk):ti,ab,kw OR (Risk Factor):ti,ab,kw OR (Social Risk Factors):ti,ab,kw OR (Factor, Social Risk):ti,ab,kw** | **126854** |
| **10** | **(Factors, Social Risk):ti,ab,kw OR (Risk Factor, Social):ti,ab,kw OR (Risk Factors, Social):ti,ab,kw OR (Social Risk Factor):ti,ab,kw OR (Health Correlates):ti,ab,kw** | **8897** |
| **11** | **(Correlates, Health):ti,ab,kw OR (Population at Risk):ti,ab,kw OR (Populations at Risk):ti,ab,kw OR (Risk Scores):ti,ab,kw OR (Risk Score):ti,ab,kw** | **103058** |
| **12** | **(Score, Risk):ti,ab,kw OR (Risk Factor Scores):ti,ab,kw OR (Risk Factor Score):ti,ab,kw OR (Score, Risk Factor):ti,ab,kw** | **44593** |
| **13** | **#8 OR #9 OR #10 OR #11 OR #12** | **187236** |
| **14** | **#1 AND #7 AND #13** | **157** |

**4.Web of science**

| **Search number** | **Query** | **Results** |
| --- | --- | --- |
| **1** | **((TS=(orthopedic trauma surgery)) OR TS=(orthopedic trauma)) OR TS=(Fracture)** | **707595** |
| **2** | **(((((((((((((((TS=(Surgical Wound Infection)) OR TS=(Infections, Surgical Wound)) OR TS=(Surgical Wound Infections)) OR TS=(Wound Infections, Surgical)) OR TS=(Infection, Surgical Wound)) OR TS=(Surgical Site Infection)) OR TS=(Infection, Surgical Site)) OR TS=(Infections, Surgical Site)) OR TS=(Surgical Site Infections)) OR TS=(Wound Infection, Postoperative)) OR TS=(Wound Infection, Surgical)) OR TS=(Infection, Postoperative Wound)) OR TS=(Infections, Postoperative Wound)) OR TS=(Postoperative Wound Infections)) OR TS=(Wound Infections, Postoperative)) OR TS=(Postoperative Wound Infection)** | **53940** |
| **3** | **((((((((((((((((((TS=(Risk Factors)) OR TS=(Factor, Risk)) OR TS=(Risk Factor)) OR TS=(Social Risk Factors)) OR TS=(Factor, Social Risk)) OR TS=(Factors, Social Risk)) OR TS=(Risk Factor, Social)) OR TS=(Risk Factors, Social)) OR TS=(Social Risk Factor)) OR TS=(Health Correlates)) OR TS=(Correlates, Health)) OR TS=(Population at Risk)) OR TS=(Populations at Risk)) OR TS=(Risk Scores)) OR TS=(Risk Score)) OR TS=(Score, Risk)) OR TS=(Risk Factor Scores)) OR TS=(Risk Factor Score)) OR TS=(Score, Risk Factor)** | **2299843** |
| **4** | **#1 AND #2 AND #3** | **1229** |

**Supplementary Material 2: Forest plot of the incidence of deep surgical site infections, sensitivity analysis and egger's test**

**
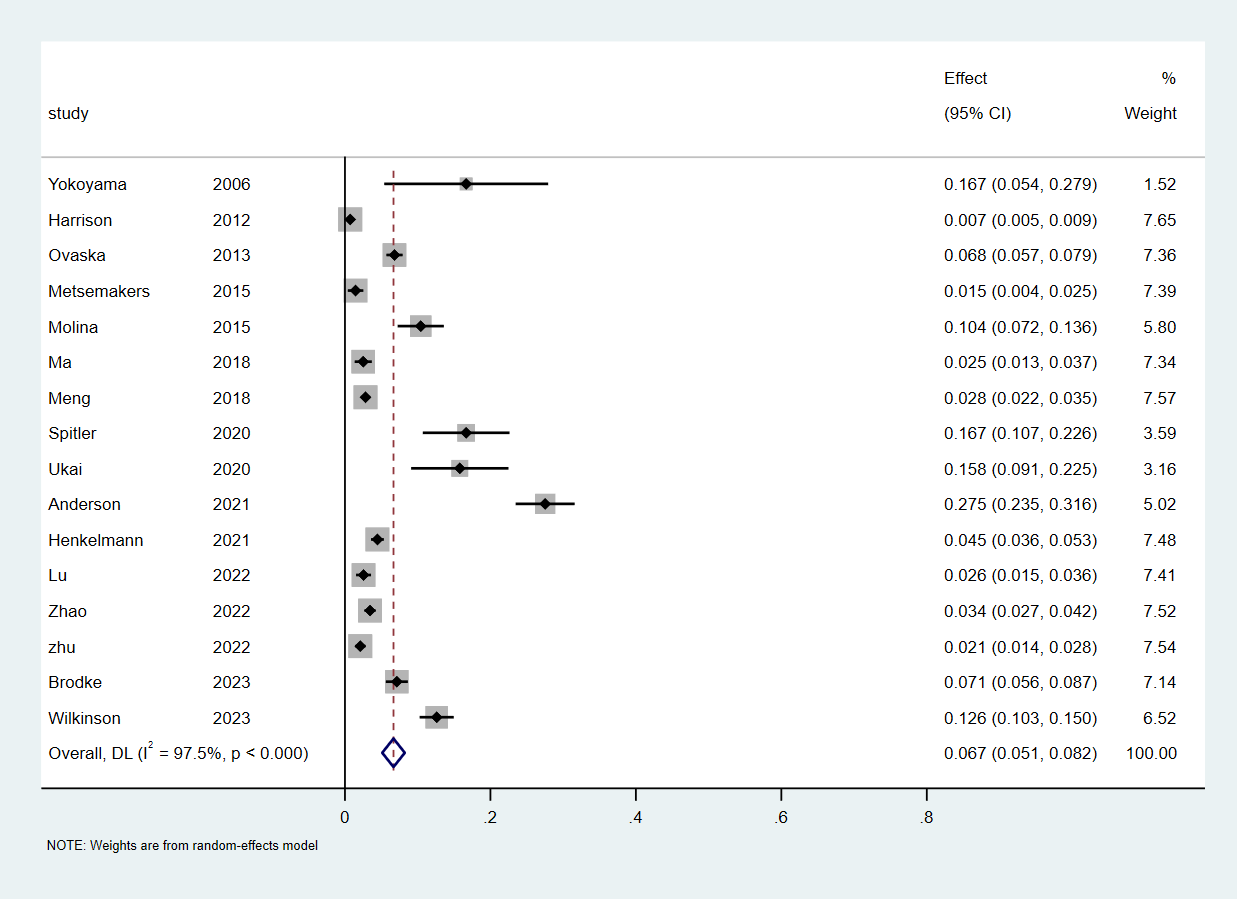
**

**
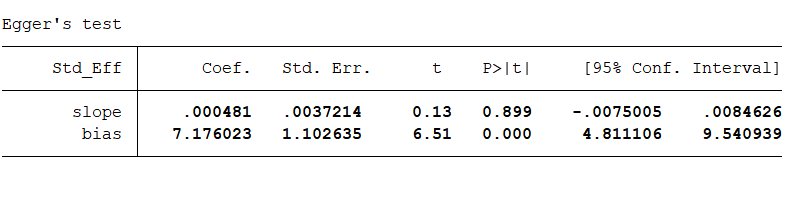
**

**
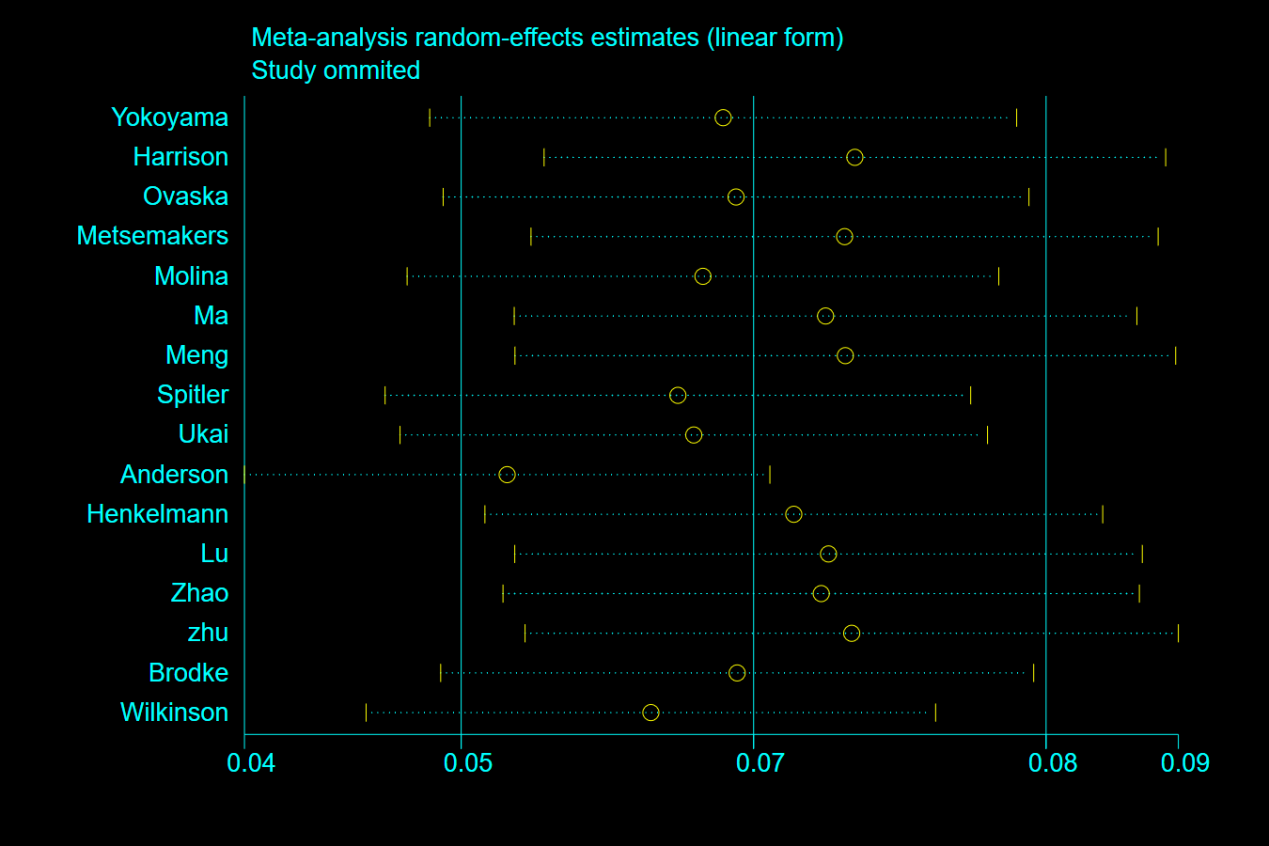
**

**Supplementary Material 3:Sensitivity analyses of risk factors**

**1.Male**

**
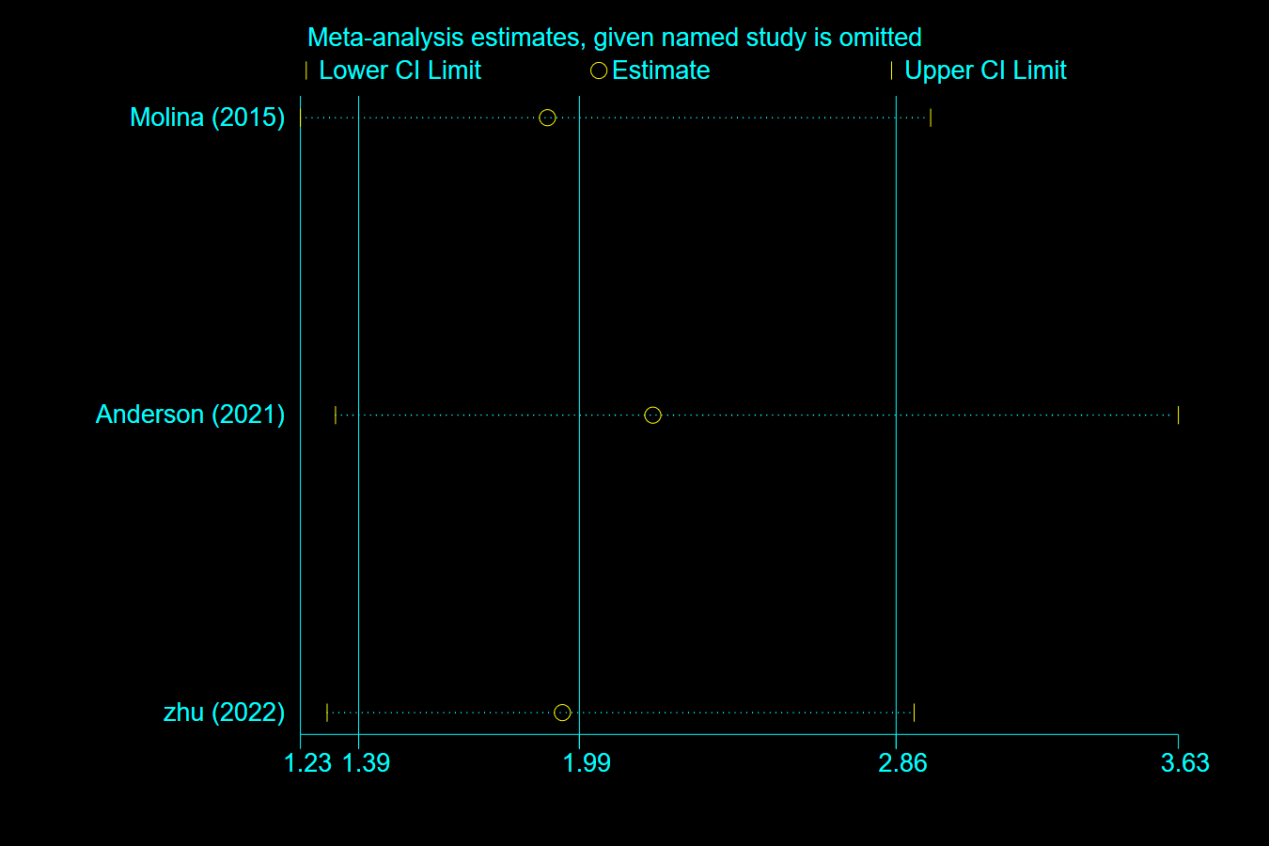
**

2.BMI


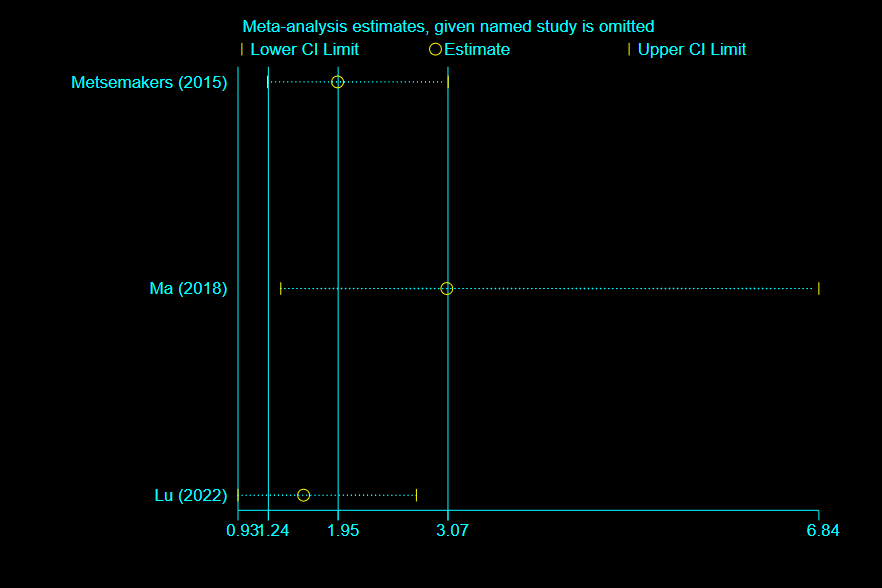


3.Current somking

**
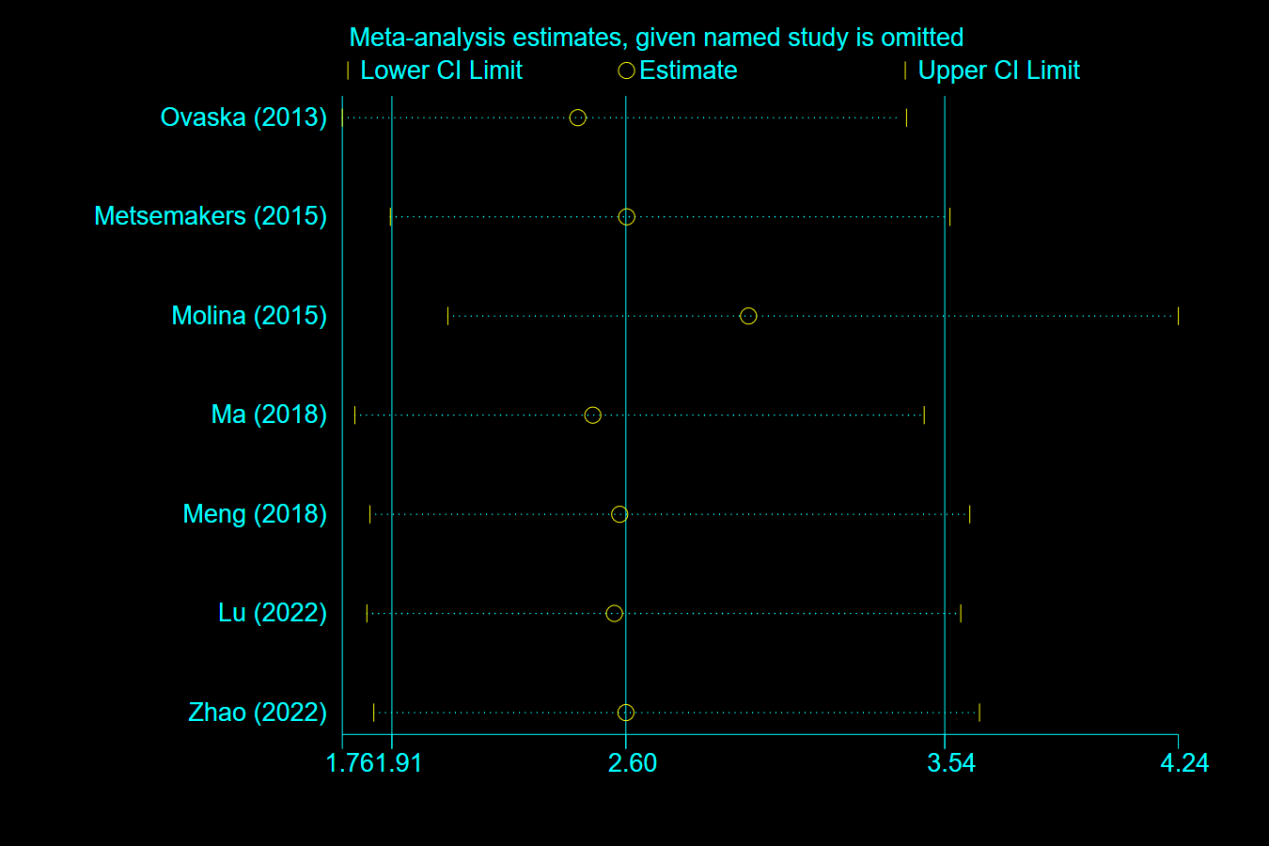
**

**4.**Wound class


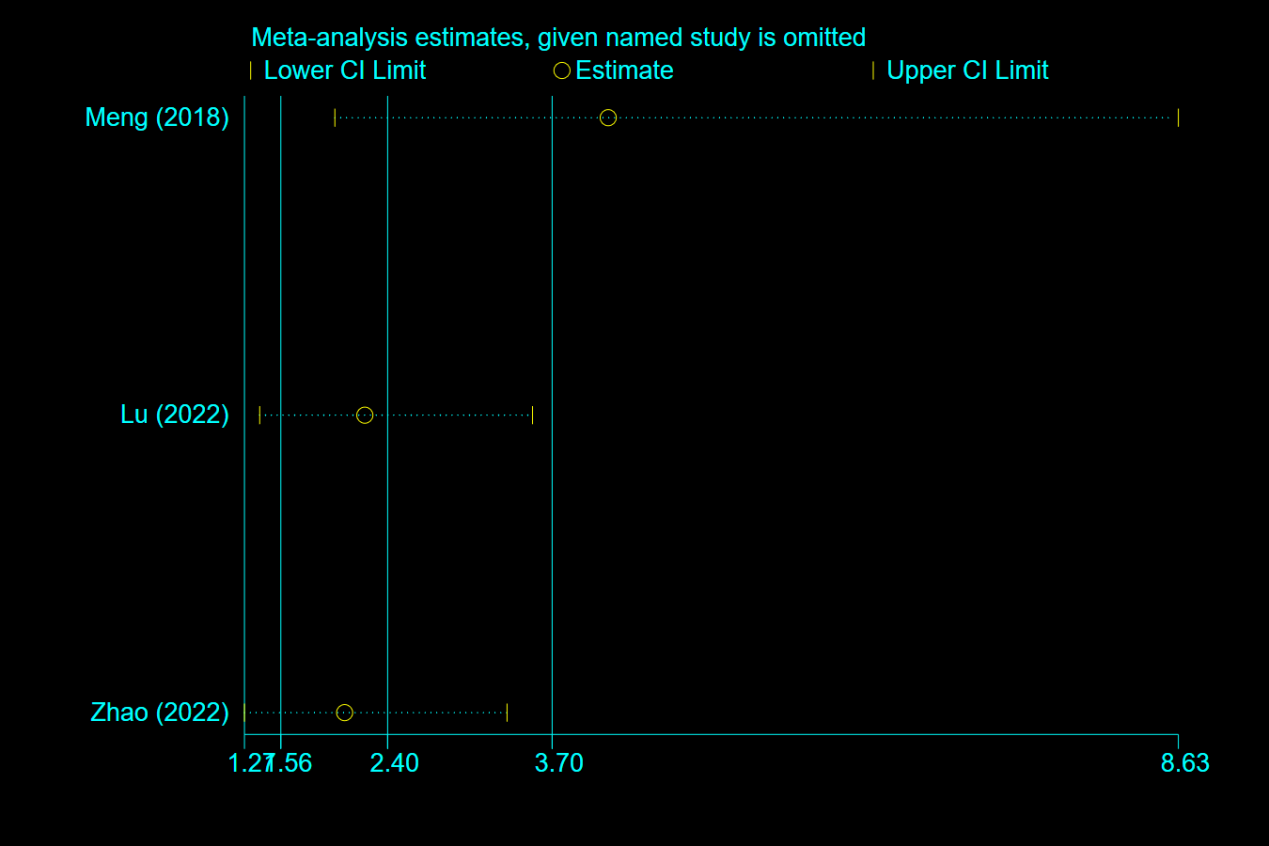


**5.**Duration of surgery


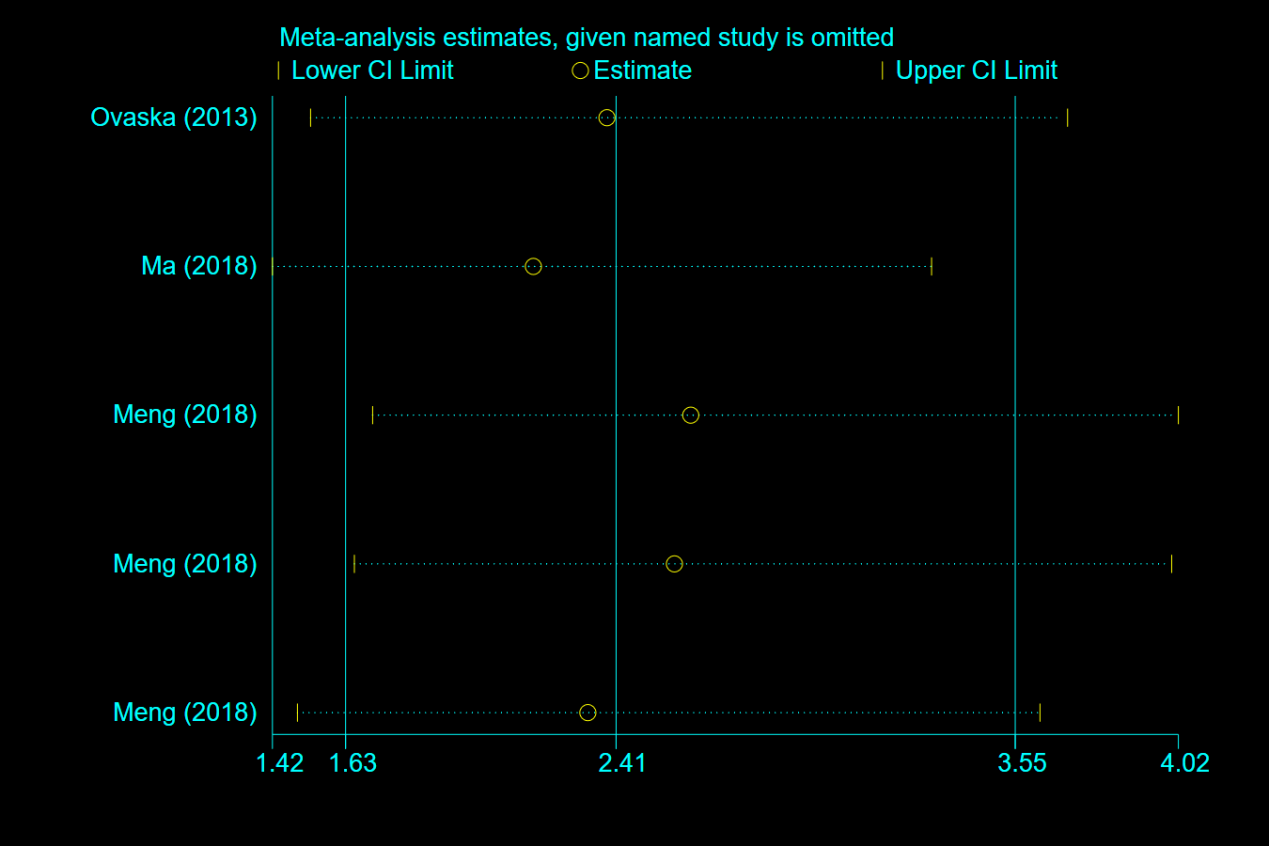


6.Open injury


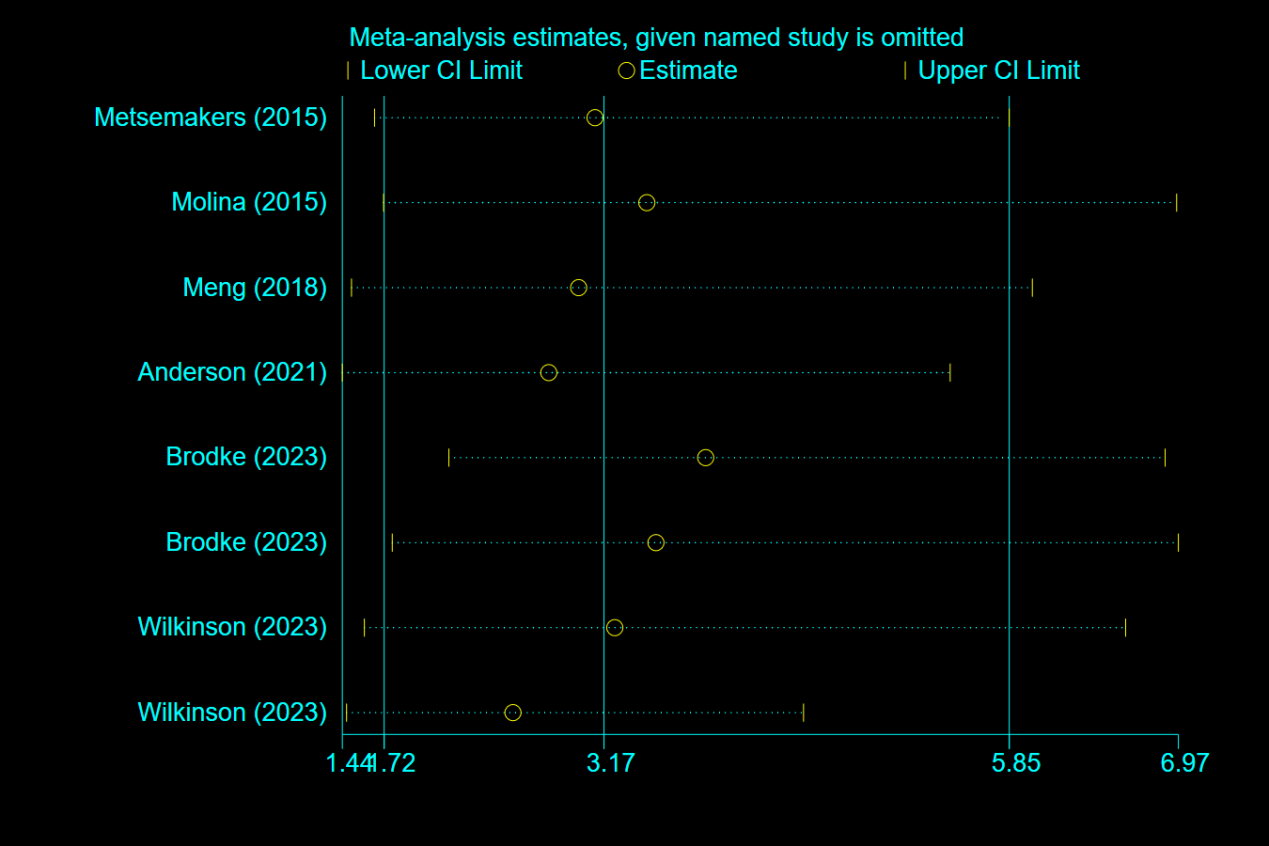

Supplement: Supplementary file 1 [file 13018_2024_5299_MOESM1_ESM.docx]
